# Wnt/β-Catenin Signaling and Obesity

**DOI:** 10.3389/fphys.2018.00792

**Published:** 2018-07-17

**Authors:** Na Chen, Jiqiu Wang

**Affiliations:** Department of Endocrinology and Metabolism, China National Research Center for Metabolic Diseases, Ruijin Hospital, Shanghai Jiao Tong University School of Medicine, Shanghai, China

**Keywords:** Wnt/β-catenin signaling, hypertrophy, hyperplasia, beige adipocytes, depot-specific

## Abstract

Obesity has become epidemic worldwide, which triggers several obesity-associated complications. Obesity is characterized by excess fat storage mainly in the visceral white adipose tissue (vWAT), subcutaneous WAT (sWAT), and other tissues. Myriad studies have demonstrated the crucial role of canonical Wnt/β-catenin cascade in the development of organs and physiological homeostasis, whereas recent studies show that genetic variations/mutations in the Wnt/β-catenin pathway are associated with human metabolic diseases. In this review, we highlight the regulation of updated Wnt/β-catenin signaling in obesity, especially the distinctly depot-specific roles between subcutaneous and visceral adipose tissue under high-fed diet stimulation and WAT browning process.

## Introduction

Worldwide obesity has more than doubled since 1980; 39% of adults aged 18 years and older were overweight in 2016, and 13% were obese throughout the world (World Health Organisation [WHO], 2017). The main reasons for the continuous rise in obesity seem to be well-known, increased food intake and decreased exercise, which result in energy consumption that exceeds energy expenditure and predispose the body to more fat accumulation.

Wnt/β-catenin signaling controls several developmental processes in early stages of life and the homeostasis of mature tissues via regulating cell proliferation (Alonso and Fuchs, 2003), differentiation (Hu et al., 2016), and genetic stability (Alberici and Fodde, 2006; Rawson et al., 2011; Hoffmeyer et al., 2012), as well as via maintenance of adult precursor cells in a pluripotent state (Sato et al., 2009). Aberration or mutation in this signaling is associated with a wide range of diseases in humans, including cancer (Porfiri et al., 1997; de La Coste et al., 1998; Anastas and Moon, 2013), osteoporosis (Gong et al., 2001; Little et al., 2002b; Loh et al., 2015), neurodegeneration (Okerlund and Cheyette, 2011; Berwick and Harvey, 2012; Inestrosa et al., 2012) and cardiometabolic disorders (Schinner, 2009). The significance of aberrant Wnt/β-catenin signaling has led to substantial efforts in the future development of therapeutic methods in metabolic disorders, including obesity. However, this field is much rough due to the enormously complex structure and a multitude of new factors involved in this pathway.

White adipose tissues (WATs) are distributed almost in the entire body and more biologically relevant with obesity. However, brown adipose tissues (BATs) principally function in the neonatal period to combat extrauterine coldness, and recent research has shown that adult humans also possess functional BAT and its mass shows a negative correlation with adiposity and body mass index (BMI) (Cypess et al., 2009, 2013; Saito et al., 2009; van Marken Lichtenbelt et al., 2009; Lidell et al., 2013; Orava et al., 2013; Chondronikola et al., 2014). Several studies have demonstrated the crucial role of Wnt/β-catenin signaling in the development of WAT and BAT, thus targeting Wnt/β-catenin pathway to combat obesity is promising.

Here we systematically reviewed literature about Wnt/β-catenin signaling and obesity, and its precise role in the browning process of depot-specific white adipocytes, to provide information about therapeutic approaches for different subgroups of the obese population.

## Association Between Obesity and Different Adipose Depots

### Overview of Obesity and Adipose Tissues

The prevalence of chronic diseases, including cancer, diabetes, and cardiovascular diseases, has been markedly increasing almost worldwide. Reducing obesity should be given priority over prevention and treatment of these chronic diseases (Heymsfield et al., 2017). The obesity crisis has led to focus on adipose tissue, which is composed mainly of adipocytes and mesenchymal cells. White and brown adipocytes in mammals have different function, morphology, and molecular features. White adipocytes are designed for energy storage in the form of fatty acids, whereas the brown adipocytes burn substrates, including fatty acids and glucose, dissipate energy, and generate heat through the process of adaptive non-shivering thermogenesis in response to various stimuli (Wang and Seale, 2016). WAT is highly plastic and dynamic, accounting for 5% to approximately 60% of body weight in different individuals, and it can change in mass in the same individual (Guo et al., 1999; Droyvold et al., 2006). Although excessive accumulation of WAT is the key feature of adiposity, obesity is clinically defined by a BMI over 30 kg/m^2^, which does not take fat content into account (World Health Organisation [WHO], 2017). Waist circumference is another index to define obesity, with a cutoff in Caucasians of 85 cm for females and 90 cm for males and in Asians 80 cm for females and 85 cm for males, which could also not distinct sWAT and vWAT proportion despite the metabolic discrepancy between them. Waist-to-hip ratio adjusted for BMI provides a surrogate measure of abdominal adiposity, which can partly reveal the association of vWAT and metabolic diseases (Emdin et al., 2017). WAT can expand via both hyperplasia and hypertrophy, and recent studies suggest that adipocyte hyperplasia is of importance to human obesity in addition to the well-known hypertrophy theory (Spalding et al., 2008; Arner et al., 2013). However, intrinsic mechanisms that lead to the increase in fat mass in response to positive energy imbalance are unknown; some details will be discussed herein.

### Differences Between sWAT and vWAT Depots

Adipose tissue comprises various discrete depots, such as inguinal, interscapular, perigonadal, retroperitoneal, and mesenteric depots, which are placed in defined positions throughout the body. There are two major divisions of WAT, vWAT, and sWAT (Cinti, 2005). vWAT and sWAT depots are simply separated based on gross anatomical location, inside or outside of the abdominal cavity. However, this gross classification indicates a variety of crucial features, including developmental lineage (Chau et al., 2014; Krueger et al., 2014; Sanchez-Gurmaches and Guertin, 2014), gene expression (Grove et al., 2010; Karastergiou and Fried, 2013; Cohen et al., 2014), adipokine secretion profiles (Shi et al., 2009), microenviroments (Jeffery et al., 2016), and metabolic characteristics (Tran et al., 2008; Karastergiou and Fried, 2013; Cohen et al., 2014).

The current prevailing view shows that sWAT and vWAT depots have different contributions to cardiometabolic disease (Emdin et al., 2017). In humans, high gluteofemoral sWAT content, which is more pervasive in premenopausal females, called “pear-shaped”, might protect against certain aspects of metabolic dysfunction (Snijder et al., 2003; Tanko et al., 2003; Manolopoulos et al., 2010; Palmer and Clegg, 2015). Conversely, high vWAT and deep abdominal sWAT depots, also known as “apple-shaped”, are thought to be correlated with increased risk of hyperlipidemia, diabetes, and cardiovascular disease (Kelley et al., 2000; Wang et al., 2005; Nicklas et al., 2006). In rodents, inguinal (posterior) sWAT is correlated with improved metabolism (Tran et al., 2008), similar to human gluteofemoral sWAT, whereas mouse perigonadal vWAT is correlated with insulin resistance (Foster et al., 2010). Most studies have demonstrated that an increased vWAT content leads to a marked degree of inflammation with macrophage accumulation (Weisberg et al., 2003), as well as adversely reduced expression of genes associated with adipogenesis and metabolism in vWAT (Nadler et al., 2000; Voigt et al., 2013; Choi et al., 2015). Hence, it has become prevalent to term sWAT depot as “good fat” and vWAT depot as “bad fat” (Berry et al., 2014). The factors that contribute to this discrepancy are still unknown.

In mice, sWAT primitive organs are formed by cells expressing adipogenic markers in comparatively late embryogenesis, and there is no obvious lipid loading of white adipocytes until birth (Birsoy et al., 2011). The visceral perigonadal WAT (pWAT) depot becomes populated after birth, with the first determined adipocyte precursors appearing at postnatal day 4 (P4) and lipid fulling in developing pWAT overt at P7 (Han et al., 2011). Moreover, recent research shows that six vWAT depots arise mainly from Wt1-expressing cells late in gestation but no sWAT or BAT from Wt1-expressing cells; these results indicate a major ontogenetic difference between vWAT and sWAT (Chau et al., 2014).

Recent progress in the identification and isolation of adipocyte precursor cells (APs, in adipose tissue, APs consist of adipocyte progenitors and preadipocytes) *in vivo* allows for direct investigation of cellular and molecular mechanisms (Rodeheffer et al., 2008; Tang et al., 2008; Lee et al., 2012; Berry and Rodeheffer, 2013; Jiang et al., 2014; Vishvanath et al., 2016). Increasing evidence suggests that APs derived from different WAT depots are distinct populations and differ in their inherent properties (Tchkonia et al., 2005, 2006; Macotela et al., 2012). The functional difference of these APs depots depends on cellular circulation, innervation, and anatomic constraints contributing to pathophysiological variation of depot-specific related metabolic homeostasis (Tchkonia et al., 2007, 2013). Of note, Tchkonia and colleagues suppose that differences between sWAT APs (S-APs) and vWAT APs (V-APs) are at least partly cell autonomous via *in vitro* APs isolated experiments (Tchkonia et al., 2013). However, a recent report indicates that instead of cell-intrinsic mechanisms, the activation of APs is regulated by the adipose depot-specific microenvironment *in vivo* (Jeffery et al., 2016). This finding highlights that APs are plastic, and both local and systemic signals could influence their differentiation potential dependent or independent of depot origin.

A variety of advances highlight the notion that definable and distinct developmental signaling cascades, including Wnt pathway, might underlie the developmental and functional discrepancy between sWAT and vWAT. Wnt signaling is critically important for health and disease, which also regulates adipose tissue in a depot-specific manner (Longo et al., 2004; Zeve et al., 2012). Therefore, understanding how depot-specific WAT mass is developmentally and functionally regulated by Wnt/β-catenin pathway *in vivo* is required.

## Overview of Wnt/β-Catenin Signaling Cascade

### Development and Structure of Canonical Wnt/β-Catenin Signaling

The Wnt/β-catenin signaling (often referred to as “canonical” signaling) was first identified in 1982 via the discovery of the Wnt1 gene (originally named Int1) in mice (Nusse and Varmus, 1982). Then the fly Wingless (wg) gene, a homolog of Wnt1, was cloned and shown that it controls proper wing formation (Baker, 1987). Epistasis experiments delineated the significance of this developmental signaling in Drosophila (Siegfried et al., 1992; Noordermeer et al., 1994; Peifer et al., 1994), and injection of mouse Int1 into early frog embryos caused body axis duplication in *Xenopus* (McMahon and Moon, 1989), both providing the highly conserved nature of the signaling, generally referred to as the canonical Wnt/β-catenin signaling. By 1996, major gaps in Wnt/β-catenin signaling were occluded with the identification of Wnt nuclear effectors, TCF/lymphoid enhancer factor (LEF) transcription factors (Behrens et al., 1996; Molenaar et al., 1996), and Wnt receptors, Frizzleds (Bhanot et al., 1996), which function with lipoprotein receptor-related proteins (LRPs)/Arrow as coreceptors (Wehrli et al., 2000).

**FIGURE 1:**
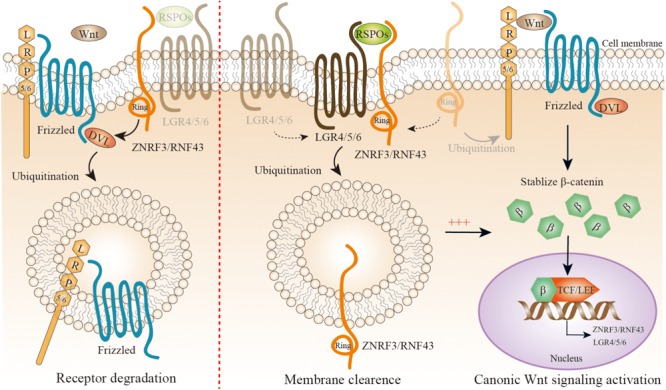
A simplified representation of precise modulation of canonic Wnt/β-catenin signaling. Wnt proteins combine with low-density lipoprotein receptor-related protein (LRP) 5 or 6 coreceptor and Frizzled (FZD) seven transmembrane domain receptors as Wnt-FZD-LRP5/6 complex; the cytoplasmic part of FZD interacts with Disheveled (DVL), and tightly regulates the activity of the β-catenin cytoplasmic pool by inhibiting its phosphorylation. Then β-catenin enters the nucleus, and turns on transcription via binding to members of the lymphoid enhancer factor (TCF/LEF) family of transcription factors. ZNRF3/RNF43 is a negative feedback regulator of Wnt/β-catenin signaling. With a lack of R-spondin proteins (RSPOs), ZNRF3/RNF43 is recruited by DVL to Wnt-FZD-LRP5/6 complex to mediate their degradation (left panel). When RSPOs are present, they simultaneously combine with the extracellular regions of both ZNRF3/RNF43 and LGR4/5/6, leading to autoubiquitination, endocytosis, and membrane clearance of ZNRF3/RNF43. Therefore, RSPOs sensitizes cells to Wnt/β-catenin signaling by inhibiting ZNRF3/RNF43, resulting in increased Wnt-FZD-LRP5/6 complex availability at the cell surface (right panel). Interestingly, both ZNRF3/RNF43 and LGR4/5/6 could be the downstream targets of Wnt/β-catenin signaling.

It is worth noting that the Wnt/β-catenin signaling is different from most other pathways. Here, we discuss the key structure of this signaling cascade via referring to several recent reviews for more details (Clevers and Nusse, 2012; Niehrs, 2012; Nusse and Varmus, 2012; Kahn, 2014; Jiang and Cong, 2016). Wnt proteins (Wnts) are cysteine-rich, secreted glycoproteins, about 40 kDa in size; they predominantly act as close-range ligands in a concentration-dependent manner to activate receptor-mediated signaling, for instance, in adult stem cell niches (Sato et al., 2011; Strand and Micchelli, 2011; Clevers and Nusse, 2012). The hallmark of this signaling pathway is that it regulates the transcriptional activity of the cofactor β-catenin (Ctnnb1), which is the core mediator of canonical Wnt/β-catenin signaling. β-catenin localizes to various subcellular sites dynamically, including adherens junctions, the cytoplasm, and the nucleus, contributing to cell–cell contacts, transcriptional regulation, and chromatin modifications (Fagotto, 2013; Miller et al., 2013). Wnts are the key extracellular regulators of β-catenin stabilization; however, there are several factors that influence β-catenin dynamics, including hypoxia, hepatocyte growth factor, prostaglandins, E-cadherin, and protein kinase A (Brembeck et al., 2006; Kawabata, 2011; van Veelen et al., 2011). Wnts combine with low-density lipoprotein receptor-related protein 5 (LRP5) or LRP6 and Frizzled (FZD) seven-transmembrane domain receptors as Wnt-FZD-LRP5/6 complex, the cytoplasmic part of FZD interacts with Disheveled (DVL) (Chen et al., 2003), tightly regulates the activity of β-catenin cytoplasmic pool by phosphorylation through inhibiting the “destruction complex”, consisting of casein kinase 1α (CK1α), glycogen synthase kinase 3β (GSK3β), tumor suppressor adenomatous polyposis coli (APC), the scaffold protein AXIN, and others (Gumbiner, 1997). Then β-catenin enters the nucleus, turns on transcription via binding to members of the lymphoid enhancer factor (TCF/LEF) family of transcription factors (**Figure 1**). In addition, β-catenin can bind to other transcription factors, thereby modulating many downstream biological processes including epithelial to mesenchymal transition, pluripotency, and melanocyte development (Le and Fodde, 2008). In the absence of Wnts, phosphorylation plays the dominant role in cytoplasmic β-catenin for its ubiquitination and proteasomal degradation (Rubinfeld et al., 1996). It is now clear there exists non-canonical Wnt signaling crosstalk with the canonical Wnt/β-catenin signaling to modulate nuclear β-catenin accumulation (Coluccia et al., 2007; Rasola et al., 2007); this review does not go into great detail about this.

### Regulation of Wnt-FZD-LRP5/6 Complex by RSPOs–LGR4/5/6–ZNRF3/RNF43 Complex

**FIGURE 2:**
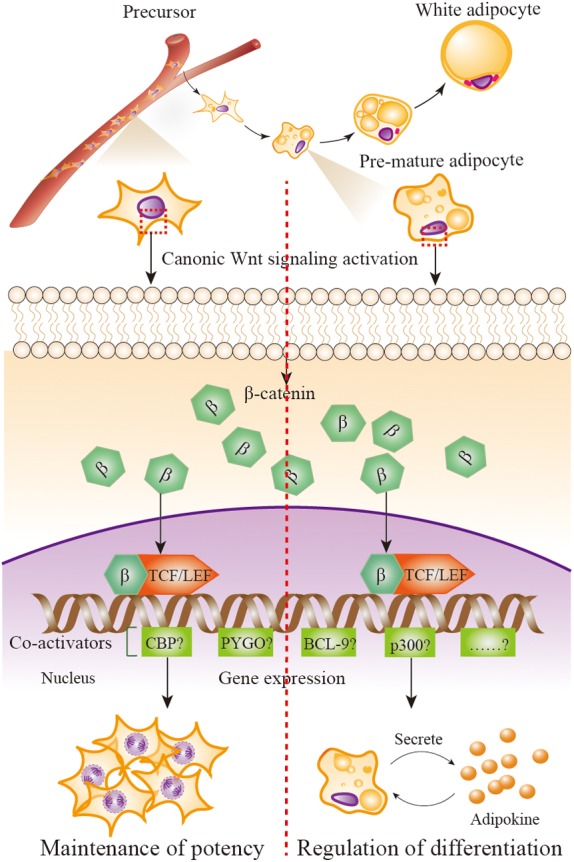
Wnt/β-catenin signaling in adipose precursors and pre-mature/mature adipocytes. When Wnt/β-catenin signaling is activated, β-catenin enters the nucleus and it turns on transcription via binding to TCF/LEF family of transcription factors. β-catenin–TCF/LEF complex is needed to recruit transcriptional co-activator to be functional, including the CREB-binding protein (CBP) or its closely associated homolog E1A associated protein p300, B cell lymphoma 9 (BCL-9), the co-activator Pygopus (PYGO), etc. Wnt/β-catenin signaling activation in adipose precursor cells leads β-catenin to combine with co-activators CBP, resulting in precursor proliferation and potency maintenance; in pre-mature/mature adipocytes, β-catenin combines with co-activator p300, resulting in the initiation of differentiation.

R-spondin proteins (RSPOs), including RSPO1-4 in mammals, are secreted proteins that amplify canonical Wnt/β-catenin signaling, but not by themselves – they only sensitize cells to Wnt/β-catenin signaling. RSPOs have a similar structure consisting of one C-terminal TSR-1 domain and two N-terminal furin repeats. The two furin repeats are pivotal for potentiating Wnt/β-catenin signaling (Kazanskaya et al., 2004; Kim et al., 2008; Li et al., 2009; Glinka et al., 2011). The TSR-1 domain might keep RSPOs close to the cell surface. LGR4/5/6 is a newly identified receptor for the RSPOs with high affinity (Carmon et al., 2011; de Lau et al., 2011; Glinka et al., 2011; Gong et al., 2012; Leushacke and Barker, 2012; Ruffner et al., 2012), and belongs to the rhodopsin G protein-coupled receptor (GPCR) family. LGRs are composed of a large 17 leucine-rich repeats extracellular domain, a hinge region, a seven-transmembrane field, and a short intracellular tail. RSPOs physically interact with LGR4/5/6, they do not activate canonical GPCR signaling (Carmon et al., 2011; de Lau et al., 2011; Ruffner et al., 2012). RSPOs–LGR4/5/6 complex is essential for amplifying Wnt/β-catenin signaling, but the way to activate the complex was unclear until the recent identification of two highly functional homologous proteins, Zinc and ring finger 3 (ZNRF3) and ring finger protein 43 (RNF43), which are the negative feedback regulators of Wnt/β-catenin signaling (**Figure 2**) (Hao et al., 2012; Koo et al., 2012). ZNRF3 and RNF43 are E3 ubiquitin ligases located on the cell surface. Their basic structure and sequence are related to the Goliath family of RING domain E3 ligases, with an extracellular protease-associated domain, a single-pass transmembrane domain, and an intracellular RING domain. ZNRF3/RNF43 inhibits Wnt/β-catenin signaling by specifically mediating the ubiquitination of the Wnt receptor FZD and coreceptor LRP6, which results in the endocytosis of Wnt receptors and their subsequent degradation. How ZNRF3/RNF43 recognizes FZD is still unknown now, and it seems that the intracellular domain of ZNRF3/RNF43 is involved in the action. Recent reports suggest that DVL is mediated as an adaptor protein combining ZNRF3/RNF43 with FZD (Jiang et al., 2015). ZNRF3/RNF43 physically interacting with DVL is required for Wnt/β-catenin signaling pathway inhibition by two proteins. Interestingly, ZNRF3/RNF43 as well as LGR4/5/6 are the downstream targets of Wnt/β-catenin, which forms an automatically negative and positive feedback loop (Jiang and Cong, 2016).

The discovery of ZNRF3/RNF43 gives a potential explanation to the molecular mechanisms of RSPOs–LGR4/5/6 complex-induced Wnt/β-catenin signaling (**Figure 1**). An initial clue to the role ZNRF3/RNF43 plays in RSPOs–LGR4/5/6 complex is that the upregulation of FZDs in cell-surface, a typical RSPOs-induced event, can be represented when ZNRF3/RNF43 lost function. This hypothesis is further supported by cell biology and biochemical experiments, in which RSPOs simultaneously combine with the extracellular regions of ZNRF3/RNF43 and/or LGR4/5/6, leading to a self-ubiquitination and endocytosis, sensitizing cells to Wnt/β-catenin signaling by increasing Wnt-FZD-LRP5/6 complex availability at the cell surface (Hao et al., 2012; Szenker-Ravi et al., 2018). This conclusion is consistent with extensive researches focusing on the RSPOs–LGR4/5/6–ZNRF3/RNF43 complex (Chen et al., 2013; Peng et al., 2013; Wang D. et al., 2013; Xie et al., 2013; de Lau et al., 2014; Zebisch and Jones, 2015). Importantly, single amino acids replacement in RSPOs completely halts the Wnts stimulatory activity of RSPOs by destructing either the RSPOs–LGR4/5/6 or RSPOs–ZNRF3/RNF43 interaction, suggesting that RSPOs need to function through interacting with both LGR4/5/6 and ZNRF3/RNF43 (Hao et al., 2012). In the complex, LGR4/5/6 works as the engagement receptor for RSPOs and ZNRF3/RNF43 works as the effector receptor. Why LGR4/5/6 are importantly required for ZNRF3/RNF43 interaction with RSPOs is not completely identified currently. It is possible that LGR4/5/6 is required for transferring RSPOs to ZNRF3/RNF43 to enhance the affinity between them (Jiang and Cong, 2016).

## Target Wnt/β-Catenin Signaling in Obesity

### Wnt/β-Catenin Signals Associate With Metabolic Disorders

During the past decade, the field involving the link between Wnt/β-catenin signaling and metabolic dysfunction is rapidly developing. The crucial role of Wnt/β-catenin signaling factors has been gradually identified by genetic and biological studies in several metabolic disorders. The first association between the Wnt/β-catenin pathway and metabolic disease came about in 2004, in which some specific SNPs in β-catenin-independent WNT5B increased susceptibility for type 2 diabetes (Kanazawa et al., 2004), initiating the discoveries of WNT10B (Christodoulides et al., 2006) and TCF7L2 (Grant et al., 2006; Tong et al., 2009; Savic et al., 2011; Takamoto et al., 2014). More than 80 signals have been found to be associated with the risk of type 2 diabetes by genome-wide association studies (Voight et al., 2010; Cho et al., 2011; Kooner et al., 2011; Morris et al., 2012; Ma et al., 2013; Mahajan et al., 2014; Steinthorsdottir et al., 2014). Among these risk loci, TCF7L2 seems to be one of the strongest risk loci [*P* = 2.1 × 10^-9^] for type 2 diabetes (Grant et al., 2006). Grant and his group have observed that heterozygous and homozygous carriers of DG10S478, within intron 3 of TCF7L2, have a higher risk with odds ratio (OR) of 1.45 and 2.41, respectively, compared with non-carriers. A recent study using genetically engineered mice reveals that TCF7L2 might be involved in glucose metabolism through regulating insulin secretion of the pancreatic beta cell mass (Takamoto et al., 2014). The direct association of TCF7L2 with obesity is unclear; however, it is well-established that TCF7L2 is expressed in adipose tissue (Cauchi et al., 2006, 2008; Kovacs et al., 2008) and involved in Wnt/β-catenin signaling dependent regulation of adipogenesis (Ross et al., 2000, 2002; Cawthorn et al., 2007). Recent research observes that TCF7L2 gene variation is associated with less weight loss during lifestyle intervention (Haupt et al., 2010) whereas Josiemer and colleagues support that TCF7L2 genetic variants may reduce body adiposity and further induce better glycemic control (Mattei et al., 2012). The specific mechanism between TCF7L2 and obesity development remains a mystery (Chen et al., 2018).

LRP5/6, is FZD coreceptor in Wnt/β-catenin signaling, the patients carrying mutations of LRP6 showed increasing risk for coronary disease and metabolic disorders (Mani et al., 2007). Previous reports suggest that patients with gain-of-function (GoF) LRP5 mutations show high bone mass (Boyden et al., 2002; Little et al., 2002a), and further study demonstrates that subjects carrying GoF LRP5 mutations and high bone mass are associated with increased lower body fat accumulation (Loh et al., 2015).

The strongest evidence for Wnt/β-catenin pathway module involved in obesity is first from the patients carrying mutations of LGR4, which is a Wnt-FZD-LRP5/6 complex coreceptor. Data from whole-genome sequencing of Icelandic individuals indicates that LGR4 heterozygous mutant subjects showed osteoporosis, electrolyte disturbance, and lowered body weight (Styrkarsdottir et al., 2013). Almost at the same time, our group discovered in humans a novel low-frequency functional missense in LGR4 (A750T), that has been linked with increased obesity risk and metabolic disorders in a study of Chinese obese patients versus controls (Wang J. et al., 2013). Alanine in 750 AAs, located between the third intracellular loop and the sixth transmembrane domain, is conserved among different species and LGRs. *In vitro* studies showed that this non-synonymous variant has gained higher biological function than wild-type LGR4, which indicates increased LGR4 activity could promote human obesity. Interestingly, constitutively activated point mutations of the corresponding site in LGR1/LHR, LGR2/TSHR, and LGR3/FSHR have been reported (Yano et al., 1995; Gozu et al., 2006; Zhang et al., 2007; Achrekar et al., 2010). In the following studies, we found that the A750T obese carriers have more severe adiposity than those obese non-carriers. Young obese carriers show increased waist circumference, waist-to-height ratio, and a remarkable increase of abdominal visceral fat area. Moreover, the carriers show increased 2-h plasma insulin and the Matsuda index, an indicator of postprandial insulin sensitivity, after an oral glucose tolerance test. These results together suggest that LGR4 might promote central obesity and its metabolic complications. Our mouse model data (Wang J. et al., 2013) has shown that compared with wild-type littermates, *Lgr4* homozygous mutant (*Lgr4*^m/m^) mice have many more beige adipocytes in vWAT, and further investigation reveals the significant increase of thermogenic genes including UCP1, PGC-1α, and Cidea. These phenomena are amplified by cold stimulation or isoprenaline treatment. Our results demonstrate that ablation of *Lgr4* in mice resists dietary and leptin mutant-induced obesity and its metabolic complications, and further prove the crucial role of LGR4 in obesity. Interestingly, recent data show that LGR2/TSHR (Draman et al., 2017) and LGR3/FSHR (Liu et al., 2017) promote browning program. It remains unknown whether the other LGRs could regulate the switch of WAT to BAT and whether they function by Wnt/β-catenin signaling. SNPs in RSPO1/RSPO3/ZNRF43 are also associated with body fat distribution. A genome-wide association studies meta-analysis identified 13 loci are in or near genes, which explains 1.03% of the variance in BMI-adjusted waist-hip ratio. Two of these, RSPO3 and ZNRF3, contribute 0.02 and 0.14%, respectively, of the variance (Heid et al., 2010). It could be concluded here that most of the factors in RSPOs–LGR4/5/6–ZNRF3/RNF43—FZDs-LRPs complex seemed to be important to body fat distribution, and their pivotal role together with Wnt/β-catenin signaling in sWAT and vWAT requires additional study.

### The Distinct Role of Wnt/β-Catenin Signaling in APs and Pre-mature/Mature Adipocytes

Zeve and his colleagues observed that altering/enhancing canonical Wnt/β-catenin signaling by transgenic and conditional GoF allele of β-catenin into APs disrupted adipose tissue development in mice. These mutant mice had a lipodystrophic phenotype, a marked lack of fat tissues, and with sequent hypertriglyceridemia. Of note, sWAT of these mice was dense with a complete loss of adipocytes, ensuring fibroblastic lineage cells derived from stem cells, whereas few remaining vWAT adipocytes were hypertrophied. In particular, the secretome of these mutant mice was also altered with increasing levels of glucodyne, a glucose-lowering hormone, which functioned similarly to insulin but through different mechanisms (Zeve et al., 2012). However, the specific origin of the postulated glucose dynamics factor is still unidentified. There are several possibilities that should be investigated, from fibroblastic lineage cells of sWAT, from hypertrophied adipocytes of vWAT, from adipose lineage cells existing in other locations, or from peripheral tissues (e.g., liver and brain), as secondary changes of the adipocyte lineage replaced by fibroblastic cells. These studies highlight the essential role of Wnt signaling in regulating depot-specific adipose precursor cells.

However, there is no systematic researches reported the role of Wnt/β-catenin signaling in the maintenance of potency of adipocyte progenitors. One study has generated NH2-terminally truncated β-catenin mice model, in which stabilized β-catenin could keep activating downstream signals, and observed an expansion of neural precursors (Chenn and Walsh, 2002). Using GSK3 pharmacological inhibitor, it has been suggested that the activation of Wnt/β-catenin signaling was involved in the maintenance of undifferentiated states of both human and mouse embryonic stem cells, along with the persisting expression of Rex-1, Oct-3 and Nanog, which are the specific transcription factors of stemness (Sato et al., 2004). Recently, more and more evidences support the notion that Wnt/β-catenin signaling plays a crucial role in pluripotency (Miyabayashi et al., 2007; Wagner et al., 2010; ten Berge et al., 2011; Clevers et al., 2014), it is not farfetched that we hypothesize this signaling also involves in the adipocyte precursor cells.

It has been proved that mature adipocytes can secrete some factors regulating adipogenesis (Janke et al., 2002). Based on our previous work and other research demonstrating an emerging role of Wnt/β-catenin signaling involved in obesity (Wang J. et al., 2013; Loh et al., 2015), it is hypothesized that Wnt/β-catenin signaling also regulates the secretion of mature adipocytes to affect metabolic function of other cells or tissues.

Why the distinct function of Wnt/β-catenin signaling in adipose precursor cells and pre-mature/mature adipocytes is uncovered till now. As mentioned previously, when β-catenin enters the nucleus, it turns on transcription via binding to members of the TCF/LEF family of transcription factors. The critical step in the activation of Wnt/β-catenin signaling is the formation of a complex comprising β-catenin and members of the TCF/LEF family (Brunner et al., 1997; van de Wetering et al., 1997). β-catenin-TCF/LEF complex needs to recruit transcriptional co-activator to be functional, including the CREB-binding protein (CBP) or its closely associated homolog E1A associated protein p300, B cell lymphoma 9 (BCL-9), the co-activator Pygopus (PYGO) (Hecht et al., 2000; Takemaru and Moon, 2000). Although the co-activators CBP and p300 are up to 93% identical and have extremely high amino acid level homology (McMillan and Kahn, 2005), a report uses the model of differential co-activator and proves the distinct functions of CBP and p300 in the Wnt/β-catenin signaling (Teo et al., 2005). That is, the utilization of CBP leads the proliferation and the maintenance of a potency transcriptional program, whereas utilization of p300 promotes a transcriptional program initiating differentiation. This outcome provides us significant clues to answer the question of whether Wnt/β-catenin signaling activation in adipose precursor cells leads β-catenin to combine with co-activators CBP, resulting in precursor proliferation and potency maintenance; whereas in pre-mature/mature adipocyte, β-catenin combines with co-activators p300, resulting in the initiation of differentiation (**Figure 2**). Of course, other possible mechanisms should not be excluded.

### Depot-Specific Adipocyte Hyperplasia and Hypertrophy in High-Fat Diet (HFD)-Induced Obesity

Although the mechanisms involved in the maintenance of proper adipocyte number and size throughout life are not known, this balance can be disrupted in obesity, leading to adipocyte hyperplasia and hypertrophy in obese models. The relative contributions of hyperplasia and hypertrophy depend on genetic factors, sex, diet risk, exposure time and duration, and the specific depot. Because differentiated adipocytes are postmitotic, adipocyte hyperplasia means an increase in *de novo* adipogenesis. Adipocyte hypertrophy is a major component of the metabolic syndrome in obese individuals (Sun et al., 2011) and commonly obeserved in obesity development, whereas individual adipocytes have a striking capacity to expand with a twofold to threefold volume (Salans et al., 1973; Hirsch and Batchelor, 1976).

Recent studies suggest that adipocyte hyperplasia plays a crucial role in the development of human obesity (Spalding et al., 2008; Arner et al., 2013). Adipocyte hyperplasia increases the risk of complications that accompany visceral obesity, including diabetes and cardiovascular disease (Wang et al., 2005; Phillips and Prins, 2008). An HFD/high-sugar diet contributes much to human obesity (Ravussin and Tataranni, 1997), and therefore, HFD-induced obesity of rodents is commonly used in the study of obesity development and its metabolic complications (Hariri and Thibault, 2010). A study using immunohistochemical analysis with the thymidine analog bromodeoxyuridine (BrdU) shows that sWAT experiences more hyperplastic growth than vWAT with HFD challenge (Joe et al., 2009). Several studies suggest that new adipocytes are formed several weeks after the initiation of HFD feeding, as the existing mature adipocytes begin to reach their maximal size (Faust et al., 1978; Joe et al., 2009). It was recently shown that the HFD greatly increases WAT mass, and the increasing size of adipocytes indicates that hypertrophy takes a large part of WAT expansion (Lee et al., 2012). In contrast to this conclusion, another study finds that, in response to prolonged HFD, the expansion of gonadal WAT (gWAT, a type of vWAT) in male mice combines both adipocyte hyperplasia and hypertrophy, whereas inguinal WAT (iWAT, a kind of sWAT) in mice does not exhibit significant adipocyte hyperplasia and mass increases mainly through hypertrophy (Wang Q.A. et al., 2013). Of note, both studies suppose that both vWAT and sWAT are first to initiate hypertrophy at the beginning of HFD. By using an adipocyte-specific, tamoxifen-inducible Adiponectin-Cre Estrogen Receptor (Adiponectin-creER) mouse model (Jeffery et al., 2014), combined with a dual fluorescent reporter, Jeffery and her colleagues performed an adipocyte pulse-chase experiment suggesting that HFD in mice rapidly and transiently increased proliferation of APs within vWAT during the first week. Yet, the newly formed APs are not immediately utilized to become mature adipocytes; they are held as a potential reserve and used after seven weeks of HFD stimulation to differentiate into new white adipocytes (Jeffery et al., 2015). These excellent advances prompt us to reflect on the mechanisms that control depot-specific adipogenesis in HFD-induced obesity.

### Wnt/β-Catenin Signaling Potential Regulation in Depot-Specific WAT Expansion of HFD-Induced Obesity

**FIGURE 3:**
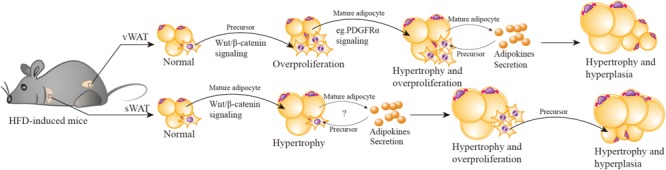
Wnt/β-catenin signaling participates in HFD-induced depot-specific hypertrophy and hyperplasia. In response to HFD stimulation, vWAT (including eWAT) adipocyte precursors are first activated by Wnt/β-catenin signaling, leading to their rapid and transient burst of proliferation “overproliferation.” Then adipocyte precursors stop “overproliferation”, remain quiescent, and wait for another signal to reactivate. Meanwhile, the PDGFRα pathway is activated, inhibits “overproliferated” adipocyte precursor adipogenesis (possibly) via suppressing Wnt/β-catenin signaling, then triggers hypertrophy of existing mature adipocytes. Hypertrophied adipocytes may secrete some adipokines to promote the subsequent lipid filling of these activated APs. However, in sWAT (including iWAT), HFD stimulation activates Wnt/β-catenin signaling to promote SWAT mature adipocytes hypertrophy. As hypertrophied adipocytes reach their maximal size and secrete adipokines or emit other signals, adipocyte precursors are then induced to “overproliferation” and new adipocytes will form to meet the demand of increasing lipid storage.

Considering the possibility that the activation of Wnt/β-catenin signaling in APs promotes their proliferation whereas in pre-mature/mature adipocytes it regulates differentiation, we hypothesize that Wnt/β-catenin signaling could play an important role in depot-specific WAT expansion (including hyperplasia and hypertrophy) of HFD-induced obesity (**Figure 3**).

Both vWAT and sWAT are composed of mature adipocytes (the fat-storing cells), adipocyte precursors (that give rise to new adipocytes), and other mesenchymal cells, which include mural, endothelial, inflammatory, and neuronal cells. In response to HFD stimulation, V-APs are first activated via Wnt/β-catenin signaling, leading to their rapid and transient burst of proliferation (“overproliferation”). Based on a previous study (Jeffery et al., 2015), the proliferation of V-APs in HFD mice is significantly decreased after one week and has no statistical difference in comparison with chow-diet mice; the proliferation phase induced by Wnt/β-catenin signaling stands a good chance during the first week. Then APs stop the “overproliferation”, remain quiescent, and wait for another signal to reactivate. Although their transformation happens at least seven weeks after HFD, the fat mass weight continues to increase during this period, indicating the contribution of hypertrophy, in which Wnt/β-catenin signaling maybe deactivated, reactivated, or linked with other mechanisms to regulate the hypertrophy. Notably, a very recent study (Marcelin et al., 2017) reports that in HFD-induced obese mice, platelet-derived growth factor receptor-α-positive (PDGFRα^+^) adipocyte progenitors can generate a fibrogenic phenotype, which is apt to vWAT fibrosis. More importantly, the activation of PDGFRα pathway promotes PDGFRα^+^ CD9^low^ cells (committed to adipogenesis) in a phenotypic shift to CD9^high^ fibrogenic cells (originate from profibrotic cells). These observations remind us of the relationship of the PDGFRα pathway and vWAT adipocyte hypertrophy. It is possible that PDGFRα pathway activation inhibits “overproliferation” of APs by suppressing Wnt/β-catenin signaling, then triggers the hypertrophy of existing mature adipocytes. It has been hypothesized that an adipogenic signal is generated to stimulate the production of new adipocytes when mature adipocytes reach their maximal size (Faust et al., 1978; Cleary et al., 1979). Wang also mentioned that because of the *in vivo* system, the maturation of activated APs takes several weeks, it is likely that the subsequent lipid filling of these APs is promoted by signal(s) from hypertrophied adipocytes (Wang Q.A. et al., 2013). The specific signal(s) is unclear; some unidentified adipokines (secreted by adipocytes) maybe present in parallel. The mechanism that controls the signal(s) activation is also unknown.

It is important to note that the manner of expansion in sWAT depot is significantly distinct from that of vWAT. The activation of Wnt/β-catenin signaling by HFD stimulation mainly promotes sWAT mature adipocytes hypertrophy, and this process is maintained for up to 12 weeks (Wang Q.A. et al., 2013). It is plausible that with a prolonged HFD stimulation exceeding 12 weeks, and hypertrophied adipocytes reach their maximal size and secrete adipokines or emit other signals, APs will be induced to “overproliferation” and new adipocytes will form to meet the increasing demand of lipid storage. To verify this hypothesis, we have ablated Ctnnb1/β-catenin specifically in mature adipocytes using Adiponectin-cre. We observed that the mutant mice resisted to long-term HFD-induced obesity and their adipocyte size was comparable to control mice, but unexpectedly, the number of mature adipocytes and APs were decreased. Further study discovered that β-catenin regulated an adipokine secretion from mature adipocytes, which could promote APs proliferation. This would provide new clues for the roles of Wnt/β-catenin in cross-talk between mature adipocytes and APs under energy enrichment environment.

During the WAT expansion process, there is crosstalk among many other possible regulators with Wnt/β-catenin signaling that control the fate of sWAT and vWAT, and the mechanism deserves more study.

## Targeting Wnt/β-Catenin Signaling in Depot-Specific “Browning”

Beige adipocytes (Ishibashi and Seale, 2010) [or brite adipocytes (Petrovic et al., 2010)], which have a multilocular morphology and express uncoupling protein 1 (UCP1) comparable to classical brown adipocytes (Wu et al., 2012; Shabalina et al., 2013), which expend energy via uncoupled mitochondrial respiration (Harms and Seale, 2013), sporadically reside within WAT (Young et al., 1984; Collins et al., 1997; Guerra et al., 1998; Cinti, 1999; Himms-Hagen et al., 2000; Vitali et al., 2012). It has been thought that beige adipocytes have no morphological difference between sWAT and vWAT depots. However, it is hypothesized that beige adipocytes of vWAT depots may have a lower content of mitochondria per cell according to a recent study (Szabo and Zoratti, 2017). Beige adipocytes can protect against the development of obesity in rodents (Seale et al., 2011), and adult humans have functional BAT mainly with beige-like adipocyte features (Sharp et al., 2012; Wu et al., 2012; Lee et al., 2014; Shinoda et al., 2015), so it becomes a novel idea to promote the WAT browning program for the treatment of obesity.

Beige adipocytes are induced by certain external cues, such as cold stimulation, β3 adrenergic receptor agonists, and exercise. This phenomenon is often called “browning” of WAT, which often occurs in the postnatal stages (Harms and Seale, 2013; Wu et al., 2013; Kajimura and Saito, 2014; Wang and Seale, 2016). The browning process involves cell fate switch and differentiation by internal hormonal, neural, and even immunological triggers and following transcription factors such as PRDM16, PGC-1α, ZNF243, Rb1, FOXC2, and EBF2 (Wang and Seale, 2016). However, the mechanisms of depot-specific beige adipocyte formation have not been completely elucidated. The two proposed mechanisms are *de novo* formation from adipocyte precursor cells and trans-differentiation of mature white adipocytes (Lee and Cowan, 2013). For example, a study has observed that adipocytes that contained “paucilocular” lipid droplets after the cold stimulation may reveal an intermediate morphological state between white and beige adipocytes (Barbatelli et al., 2010). Moreover, a recent study uncovered a fundamental phenomenon that after withdrawing external stimuli, beige adipocytes progressively lose their morphological and molecular characteristics, directly acquiring white-like characteristics bypassing an intermediate precursor stage (Altshuler-Keylin et al., 2016). This is consistent with a previous study (Rosenwald et al., 2013) using a genetic UCP1 lineage-tracing method, showing that after five weeks of warm adaptation, activated beige adipocytes of sWAT would convert into mature white adipocytes; importantly, these white-type adipocytes had the capacity to reconvert into beige adipocytes when re-exposed to cold stimulation. This study also proposed that there may be dormant beige adipocytes (unilocular and UCP1^-^) that can be active to multilocular and UCP1^+^ beige adipocytes. Interestingly, a new study found that beige adipocytes of vWAT could clearly be divided into two distinct subpopulations, UCP1^+^ and UCP1^-^ (Bertholet et al., 2017). However, the work of Wang observed that most of the newly emerging beige adipocytes were derived from *de novo* differentiation of precursors in sWAT by performing pulse-chase fate-mapping experiments of mature adipocytes (Wang Q.A. et al., 2013). Hence, the specific origin of beige adipocytes still needs to be critically investigated by monitoring the life cycle dynamics of beige adipocytes at a single-cell resolution, and this review does not go into great detail about this.

Previous studies have suggested that the appearance of beige cells is observed mainly in sWAT, and newly emerging beige cells are hardly detected in the vWAT depot (Seale et al., 2008). The most significant functional difference between S-APs and V-APs is that the former has a higher proliferation rate and better differentiation than the latter in response to adipogenic stimuli *in vitro* (Tchkonia et al., 2005; Macotela et al., 2012). However, several works (Himms-Hagen et al., 2000; Lee et al., 2012; Frontini et al., 2013) found that an increase of the mitotic index of V-APs was far greater than S-APs in response to the treatment of CL316,243, a β3-adrenergic receptor agonist. Most newly emerging beige adipocytes in vWAT were positive for BrdU or Ki67; however, only a small percentage of indicated beige adipocytes in sWAT were BrdU^+^ or Ki67^+^.

**FIGURE 4:**
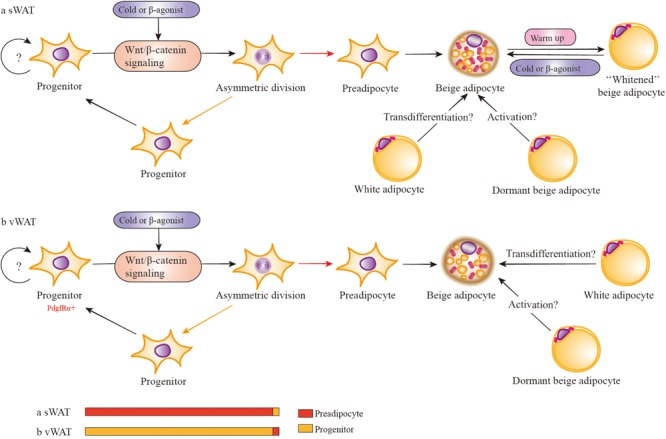
Wnt/β-catenin signaling regulates the development of depot-specific beige adipocytes. In response to cold or β3 adrenergic receptor agonists, both sWAT and vWAT adipocyte progenitors (in adipose cells, APs consist of adipocyte progenitors and preadipocytes) are induced to asymmetric division possibly via the activation of Wnt/β-catenin signaling. However, the division percentage between preadipocytes and adipocyte progenitors is different in sWAT and vWAT. Most likely, the percentage of vWAT adipocyte progenitors dividing into progenitors is far more than that of preadipocytes. There is the possibility that mature white adipocytes may directly transform into beige adipocytes via transdifferentiation *in vivo*. In addition, there are dormant beige adipocytes (unilocular and UCP1^-^) that can be activated to multilocular and UCP1^+^ beige adipocytes. Activated beige adipocytes of sWAT can be converted into white-type adipocytes when warmed up, and these white-type adipocytes have the capacity to reconvert into beige adipocytes when re-exposed to cold stimulation.

It is clear that Wnt/β-catenin signaling has a crucial role in the commitment of maintenance and induction of differentiation. The study by Michael (Kahn, 2014) has depicted the model of stem cell division, including symmetric and asymmetric division, and described how the outcome of the division is regulated by mitotic spindle. This model leads us to consider the possibility that Wnt/β-catenin signaling activates the depot-specific APs asymmetric division (**Figure 4**).

In response to cold or β3-adrenergic receptor agonists or other beige adipogenic stimulations, both sWAT and vWAT adipocyte progenitors are induced to asymmetric division via some crucial signals, such as the activation of Wnt/β-catenin signaling. However, the division percentage into adipocyte progenitors and preadipocytes is different in sWAT and vWAT. Most likely, the percentage of vWAT adipocyte progenitors dividing into progenitors is far more than preadipocytes, resulting in the majority of newly emerging beige adipocytes being positive for BrdU (Lee et al., 2012). However, the percentage is lower in sWAT adipocyte progenitors, which divide into progenitors much less often than preadipocytes, and the lack of BrdU^+^ cells (Lee et al., 2012) indicates the absence of the cell-proliferative step in sWAT. If beige adipocytes are derived from differentiated preadipocytes with very limited proliferation of progenitors, the newly emerging cells will also be BrdU^-^. In addition, it is consistent with previous observations that induced beige cells are mainly found in sWAT and are seldom detected in vWAT (Seale et al., 2008). Regarding previous studies about Wnt/β-catenin signaling with adiposity, it is important to demonstrate the role of RSPOs-LGRs-ZNRF3/RNF43-FZDs-LRPs complex in asymmetric division in depot-specific adipocyte progenitors during browning.

## Conclusion

What is the precise role of Wnt/β-catenin signaling in depot-specific browning and white fat expansion? Which factors interact with Wnt/β-catenin signaling underlying the different features of sWAT and vWAT? What is the mechanism by which Wnt/β-catenin signaling maintains the potency of APs and regulates the differentiation of mature adipocytes under external stimuli? If beige cells are derived from preadipocytes, the question is how Wnt/β-catenin signaling regulates the preadipocytes differentiation into beige cells. And if there are other sources for beige cells, such as trans-differentiation from mature adipocytes and activation of dormant beige adipocytes, how does Wnt/β-catenin signaling be involved in these processes, and how does it participate in and are there different roles and mechanisms in different WAT depots? Although we cannot yet definitively answer these questions, according to more and more evidences and our recent interesting discovery about the role of β-catenin in mature adipocytes, it is highly hypothesized that Wnt/β-catenin signaling is crucial for obesity development, including adipocyte hyperplasia, hypertrophy, browning, and even apoptosis, in a depot-specific manner.

With the application of new methodologies and experimental models to the field of adipose tissue biology, we have the opportunity to definitively explore the WAT intrinsic mechanisms that regulate adipocyte expansion and browning *in vivo*. As the pathogenesis, clinical features, and intervention outcomes of obesity are greatly affected by fat distribution and cellular characteristics, the study of Wnt/β-catenin signaling in WAT expansion and browning *in vivo* has the potential to discover novel therapeutic targets for precise clinical stratification and treatment for obesity.

## Author Contributions

JW and NC conceived the project. NC drafted the paper. JW contributed comments and advice on the manuscript. All authors were involved in editing the manuscript.

## Conflict of Interest Statement

The authors declare that the research was conducted in the absence of any commercial or financial relationships that could be construed as a potential conflict of interest. The reviewer DV and handling Editor declared their shared affiliation.
